# Childhood-onset ocular mucous membrane pemphigoid presenting with peripheral ulcerative keratitis: a case report and review of the literature

**DOI:** 10.1186/s12348-025-00480-y

**Published:** 2025-05-29

**Authors:** Eugenia M. Ramos-Dávila, Raul E. Ruiz-Lozano, Alejandro Rodríguez-García, Carlos Álvarez-Guzmán

**Affiliations:** 1 Tecnologico de Monterrey, Escuela de Medicina y Ciencias de la Salud, Monterrey, Mexico; 2 Bascom Palmer Eye Institute, University of Miami, Miami, FL USA; 3 Institute of Ophthalmology and Visual Sciences, Av. Batallon de San Patricio #112. Col. Real de San Agustin, Monterrey, N.L. CP. 66278 Mexico

## Abstract

**Purpose:**

The purpose of this study was to describe the case of a pediatric patient diagnosed with mucous membrane pemphigoid (MMP) with exclusive ocular involvement presenting at diagnosis with peripheral ulcerative keratitis and provide a review of the literature.

**Methods:**

A 12-year-old girl presented with cicatricial conjunctivitis and peripheral ulcerative keratitis (PUK). A conjunctival biopsy and direct immunofluorescence revealed linear deposits of IgG, IgM, and C3 at the basement membrane zone, confirming a diagnosis of ocular MMP.

**Results:**

The patient was initially treated with dapsone 25 mg/day and prednisone 40 mg/day along with topical treatment including cyclosporine 0.05%, loteprednol etabonate 0.05%, and sodium hyaluronate 0.15% and trehalose 3%. Inflammation persisted as an increase in the extension of symblepharon was documented. Subsequently, dapsone was switched to oral methotrexate 15 mg/week and prednisone was successfully tapered to 5 mg/day. After three years of follow-up, disease activity remained quiescent.

**Conclusions:**

Pediatric mucous membrane pemphigoid with ocular involvement is a rare condition of which few reports have been published, resulting in scarce information regarding its clinical course and response to treatment. We report the first case observed in a Hispanic patient, opening with peripheral ulcerative keratitis, and responding successfully to methotrexate.

## Introduction

Mucous membrane pemphigoid (MMP) is a rare systemic cicatrizing autoimmune disease that primarily affects the skin but more commonly involves mucous membranes, such as the conjunctiva, nasal cavity, oropharynx, esophagus, trachea, skin, and genitalia [1]. It has an estimated annual incidence of 2 cases per million, exhibiting a pronounced predilection for females, Caucasians, and elderly patients with a mean age at onset of 60–65 years [1, 2].

Ocular involvement is observed in up to 70% of patients, leading to vision loss in nearly 50% of cases. This outcome is attributed to chronic progressive sub-epithelial fibrosis, tissue remodeling, and neovascularization [2]. Regrettably, preventable progressive fibrosis develops in nearly 40% of patients as the diagnosis is frequently delayed due to the non-specific nature of early clinical symptoms and signs and the limited sensitivity of immunopathological studies [3]. Previous reports indicate that only 50% of patients with ocular MMP present a positive direct immunofluorescence (DIF) result and that 26% exhibit false negative results, requiring multiple biopsies [4]. Prompt and aggressive treatment with immunosuppressive therapy is advocated; however, severe cases may progress and cause substantial morbidity despite intervention [1].

Based on the available information, only five pediatric cases have been documented in which MMP manifested exclusively ocular symptoms. These cases pose not only a diagnostic challenge but also a therapeutic dilemma since immunosuppressive drugs carry a considerable number of adverse effects, which is a matter of particular concern in children. We report the case of a 12-year-old girl diagnosed with ocular MMP and provide a review of previously reported cases of the disease appearing early in life.

## Case report

**Fig. 1:**
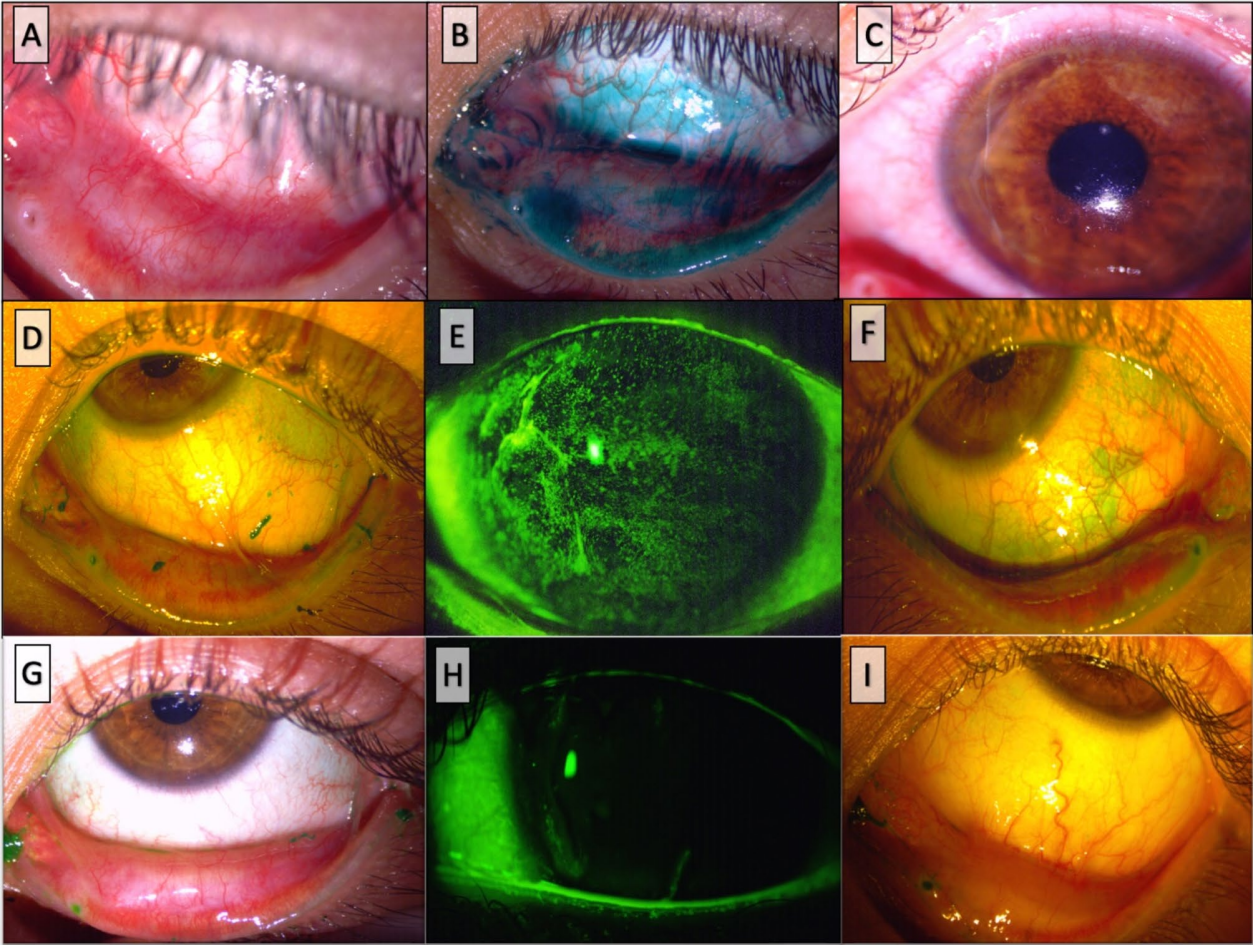
Clinical images illustrating the progression observed from the initial presentation. (**A**, **B**,) Subepithelial fibrosis, symblephara, and lissamine green staining in the bulbar and palpebral conjunctiva at first presentation. (**C**) Peripheral ulcerative keratitis on the left eye presenting two weeks after initial treatment. (**D**, **G**, **F**) Worsening of lissamine green staining, symblephara, and punctate epithelial keratitis. (**G**, **H**, **I**) Inflammation subsides in both the conjunctiva and cornea after treatment with methotrexate

**Fig. 2:**
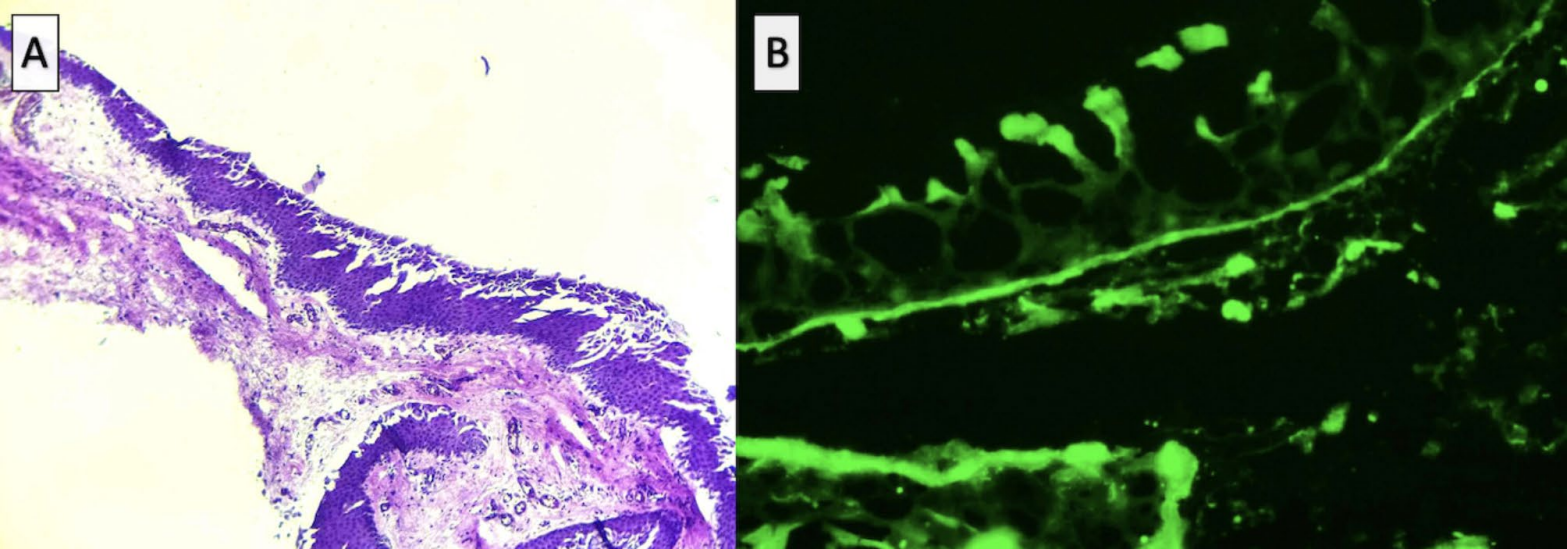
Findings from the histopathological and immunohistochemical examination of the conjunctival biopsy. (**A**). Histopathological results of the conjunctival biopsy demonstrate non-keratinized stratified squamous epithelium with areas of acantholysis and scant chronic vascular inflammatory infiltrate. (**B**) Direct immunofluorescence displays a linear pattern of IgG, IgM, and C3 deposits on the basal lamina

An otherwise healthy twelve-year-old female was referred to the Ophthalmology Department at Tecnologico de Monterrey, presenting with subacute redness in the left eye. The parents reported a 5-year history of chronic blepharitis and allergic conjunctivitis. Upon ophthalmic examination, a best corrected visual acuity (BCVA) of 20/25 and 20/40 was found in the right (OD) and left (OS) eye, respectively. A slit lamp exam revealed mild meibomian gland, dysfunction, and diffuse superficial punctate keratitis in both eyes (OU) accompanied by moderate conjunctival hyperemia in OS. Additionally, sub-epithelial fibrosis and symblepharon were observed in the nasal and temporal quadrants of the lower conjunctiva OU. (Figure 1A and B). Notably, no papillary or follicular reactions were observed. The anterior segment, intraocular pressure, and fundoscopy yielded unremarkable findings. The patient denied symptoms of dry mouth or dysphagia, and no dermatologic lesions were observed. Loteprednol etabonate 0.5% four times daily, sodium hyaluronate 0.15% and trehalose 3% every 6 h, lid hygiene, and topical azithromycin 15 mg/g solution once a day were prescribed as initial treatment. The patient returned two weeks later with severe pain and decreased BCVA of 20/400 in OS. A temporal corneal stromal thinning was observed in the same eye (Fig. 1C), establishing the diagnosis of peripheral ulcerative keratitis (PUK). Treatment was adjusted to include prednisone 20 mg/day and combined moxifloxacin 0.5%/ dexamethasone 0.1% eyedrops every 4 h. After one week under this therapeutic regimen, the symptoms and vision improved to 20/50 in the left eye. Laboratory tests including a complete blood count, antineutrophil cytoplasmic antibodies (ANCA), antinuclear antibodies (ANA), rheumatoid factor, Anti-Ro/SSA and Anti-La/SSB antibodies, blood levels of complement components C3 and C4, C-reactive protein, and erythrocyte sedimentation rate were ordered, and biopsy from the superior and temporal bulbar conjunctiva in OS was performed. Results from laboratory tests were unremarkable except for decreased levels of serum complement fraction C4 at 4.0 IU/ml. The biopsy results from the upper bulbar conjunctiva revealed a non-keratinized stratified squamous epithelium with areas of acantholysis and scant chronic vascular inflammatory infiltrates (Fig. 2A). Linear deposits of IgG, IgM, and C3 were detected with DIF in the basement membrane zone (BMZ), consistent with the diagnosis of ocular MMP (Fig. 2B). Topical cyclosporine 0.05% twice a day was included in the treatment regimen [5]. Following the confirmation of blood levels of glucose-6-phosphate dehydrogenase within range, dapsone 50 mg/day was initiated. However, inflammation persisted for the following three months and an increase in the extension of symblepharon in OS was documented, warranting the decision to switch from dapsone to oral methotrexate at 15 mg/week, due to gastrointestinal side effects, and increase oral prednisone to 40 mg/day (Fig. 1C, D and E). Remarkable clinical improvement was noted two months following the treatment modification, leading to the successful taper of oral prednisone. (Figure 1F, G and H).

At the last follow-up visit, twenty-eight months after the initial consultation, ocular inflammation remained quiescent and discomfort subsided; however, severe meibomian gland dysfunction persisted. The patient remains on a treatment regimen that includes oral methotrexate 15 mg/week with folic acid supplementation, topical cyclosporine 0.05% twice a day, loteprednol etabonate 0.05% once a day, sodium hyaluronate 0.15% and trehalose 3% every 6 h, dexpanthenol 5% every night, and prednisone 5 mg/day.

## Discussion

Childhood-onset MMP is a rare condition, documented in only 28 patients reported in the literature, including mostly females (*n* = 19, 68%) with a median age at diagnosis ranging from 1 to 17 years [6]. However, an estimated delay in diagnosis of 2 months to 5 years has been recorded among children with the disease. The most frequently affected sites in pediatric patients included the oral cavity (*n* = 18, 64%), followed by the conjunctiva (*n* = 11, 39%), the genital mucosa (*n* = 10, 35%), the skin (*n* = 6, 21%), and the upper respiratory pathway (*n* = 5, 62.5%) [7–9]. Notably, only five patients presented isolated ocular disease [10–13]. Moreover, all patients with skin affection also presented ocular manifestations [6–9, 14–16]. Two patients with oral mucous, skin, and ocular affection died; the cause of death was not available for one while the other patient died from tracheal stenosis associated with the disease [15, 16]. General characteristics of previously reported cases of childhood-onset MMP are displayed in Tables 1 and 2. Likewise, tissue involvement in adults presents most commonly in the oral cavity (85%), followed by the conjunctiva (65%), nasopharynx (20–40%), skin (20–35%), genitalia (20%), and more rarely the esophagus and trachea (5–15%) [17]. The prevalence of affected sites and features appears to be comparable to that of pediatric patients; however, genital involvement in children seems to be more prevalent than in adults.

**Table 1:** Previously reported cases of childhood-onset MMP with ocular involvement

Reference	Age (years)/sex	Systemic findings	Delay in diagnosis	Treatment	Commentary
Jolliffe et al., 1977	13/girl	Oral, genital, skin	3-months	Tetracosactid Depot 1 mg IM twice a week + Prednisolone 90 mg/day	None
Rosenbaum et al., 1984	6/boy	Oral, genital, skin	1-year	Methylprednisolone IV + Dapsone 2 mg/kg/day	Response to first-line treatment
Iglesias L et al., 1992	12/boy	None	5-years	Dapsone	None
Kanwar et al., 2006	14/girl	Nasal, oral, laryngeal	6-months	Prednisolone 30 mg/day + Dapsone 100 mg/day	Coeliac disease
Gamm D et al., 2006	17/girl	Oral, tracheal, skin	1-year	Prednisone + dapsone	Deceased from tracheal stenosis
Iovine A et al., 2008	9/boy	Oral, genital, laryngeal, skin	5-years	Prednisolone 25 mg/day + Cyclosporine	Bilateral corneal perforation Reconstructive surgery Deceased
Kharfi M et al., 2010	1/boy	Oral, nasal, skin	10-months	Prednisone 2 mg/kg/day + Dapsone 25 mg/day for 3 years + topical cyclosporine every 6 h for 3 years	Bilateral corneal opacities
Levallee A et al., 2013	12/girl	None	2-years	Dapsone 100 mg/day	PUK episode Relapse after withdrawal
Flores-Climente et al., 2019	2/boy	None	6-months	1. Topical cyclosporine 2. Prednisolone 1 mg/kg/day + MMF 15 mg/kg/day + cyclosporine 4 mg/kg/day 3. Dapsone 2 mg/kg/day 4. Rituximab 375 mg/m2 at 2-week intervals	Responded to biologic therapy
Pattnaik M et al., 2019	14/girl	Oral, genital, nasal, skin	6-months	1. Prednisolone 1 mg/kg/day + Dapsone 2 mg/kg/day 2. Azathioprine 100 mg/day	Responded to second line treatment
Ollero et al., 2022	1/boy	None	5-months	1. Topical cyclosporine + botulinum toxin 2. Prednisolone steaglate + MMF 3. Cyclosporine 4. Dapsone 5. Rituximab	Responded to biologic therapy Required reconstructive surgery
Jitender MS et al., 2024	13/girl	None	1-year	1. Prednisolone 0.5 mg/kg/day + dexamethasone 0.1% eyedrops 2. Azathioprine 50 mg/day	Well-controlled
Jitender MS et al., 2024	16/girl	Oral	2-years	Prednisolone 0.5 mg/kg/day + dexamethasone 0.1% eyedrops and azathioprine 50 mg/day	Residual corneal scarring
Ramos-Dávila et al., 2024	12/girl	None	4-years	1. Prednisone 20 mg/day + topical cyclosporine + dapsone 25 mg/day 2. Prednisone 40 mg/day + Methotrexate 20 mg/week + topical cyclosporine	Responded to methotrexate

*MMF, mycophenolate mofetil; mg, milligrams; kg, kilograms; m^2^, square meters; IM, intramuscular; IV; intravenous; PUK, peripheral ulcerative keratitis

**Table 2:** Previously reported cases of pediatric mucous membrane pemphigoid without ocular involvement

Reference	Age (years) / Sex	Affected sites	Delay in diagnosis	Treatment	Response
Rogers et al., 1981	4/girl	Genitalia	8-months	NA	NA
Barnett et al., 1981	13/boy	Gingivitis	9-months	Non-specific steroids	Yes
Moy et al., 1986	9/girl	Gingivitis	4-years	Prednisone 40 mg/day	Yes
Laskaris et al., 1988	14/girl	Gingivitis	2-years	Topical 0.1% triamcinolone paste	Yes
Sklavounou et al., 1990	13/boy	Gingivitis	1-year	Topical 1.0 mg betamethasone/day	Yes
Roche C et al., 2009	14/girl	Gingivitis	4-month	Mouth rinse with triamcinolone and chlortetracycline + miconazole oral gel	Yes
Farrell et al., 1999	14/girl	Genitalia	9-months	Topical clobetasol propionate cream	Yes
Farrell et al., 1999	8/girl	Genitalia and gingivitis	NA	Prednisolone + dapsone + sulphapyridine	Yes
Cheng et al., 2001	8/girl	Gingivitis	6-months	Mouth rinse with 0.05% fluocinonide + 0.2% chlorhexidine	Yes
Schoeffler et al., 2004	9/girl	Genitalia	4-months	Clobetasol propionate cream + dapsone 1.5 mg/kg/day	Yes
Hoque et al., 2005	7/girl	Genitalia	9-months	Clobetasol propionate cream	Yes
Lourenco et al., 2006	4/girl	Gingivitis	2-years	Dapsone 50 mg/day for 20 months	Yes
Musa et al., 2002	9/girl	Gingivitis	8-months	Topical 0.05% fluocinonide gel switched to topical betamethasone. Poor compliance.	Poor
Lebeau et al., 2004	8/girl	Genitalia	5-years	Topical tacrolimus 0.1% for 9 months	Yes
Mostafa et al., 2009	6/boy	Gingivitis	1-year	Betamethasone cream and 0.2% chlorhexidine wash switched to 0.05% fluocinonide	Yes

*NA, not available; mg, milligrams; kg, kilograms

The diagnosis is challenging, compounded by the very low prevalence of the disease in pediatric patients. In this population, reaching diagnosis could be hampered by alternative etiologies for cicatricial conjunctivitis such as hemorrhagic adenoviral conjunctivitis with membrane formation, chlamydial /trachoma keratoconjunctivitis, severe vernal keratoconjunctivitis, and pediatric blepharokeratoconjunctivitis, among others [18]. Moreover, patients may present with a long history of using diverse and multiple topical eye drops and potentially be misdiagnosed as drug-induced conjunctivitis. Proper diagnosis requires concordance between clinical signs and the detection of anti-basement zone autoantibodies. These autoantibodies are tissue-bound, detected by DIF microscopy, or circulating when detected by indirect immunofluorescence (IIF) [17]. DIF showing linear IgG, IgA, and/or C3 deposits at the subepithelial BMZ and/or dermal-epidermal junction is strongly recommended as the major single diagnostic test for MMP [17, 19]. Nonetheless, inaccurate tissue management or collection, along with initially negative results during the early stages of the disease, can result in false-negative outcomes, thereby impeding the timely and accurate diagnosis [3, 11, 17]. Likewise, a previously reported case of ocular MMP in a 12-year-old girl presented a negative DIF result at the initial presentation, subsequently turning positive two years later [11]. The mentioned case was the only patient who manifested an episode of PUK, as in our report. Autoimmune diseases are responsible for nearly half of the non-infectious etiologies of PUK, with rheumatoid arthritis and granulomatosis with polyangiitis being the most frequently underlying diagnoses [20, 21]. Moreover, PUK has only been documented in two additional patients with MMP; the before-stated pediatric patient and an older adult woman developing PUK following cataract surgery [11, 22].

After diagnosis has been properly established, prompt treatment should be initiated. Dapsone (1.0-1.5 mg/kg/day) combined with oral cyclophosphamide (2 mg/kg/day), or corticosteroids (0.5–1.5 mg/kg/day) are the recommended treatment options in adults diagnosed with ocular MMP [5, 17]. Cyclophosphamide has been particularly associated with a prompt response and prolonged remission in patients with ocular involvement [17]. Other alternatives include azathioprine (1.5-2.0 mg/kg/day), methotrexate (5–25 mg/week) [23], mycophenolate mofetil (2 g/day), or sodium mycophenolic acid (1,440 mg/day) [17, 24]. Biologic therapy, such as rituximab (375 mg/m^2^ weekly for 4 weeks or 1,000 mg twice every 2 weeks) is usually reserved for refractory cases of severe MMP [17, 25]. Treatment in children is hindered by the extensive side effects of the drugs mentioned above and the lack of standardized regimens for MMP in this demographic. Prior cases of pediatric MMP included cyclosporine (4 mg/kg/day) in the treatment regimen; however, two out of three patients receiving this drug were unresponsive and required switching to another agent [12, 13, 15]. Interestingly, none of the prior cases were treated with methotrexate, an antimetabolite extensively studied in the pediatric population for other immune-mediated diseases, rendering an acceptable safety profile [23, 24, 26, 27]. The patient in our report presents the first case of pediatric ocular MMP successfully treated with methotrexate (15 mg/week). Importantly, the long-term use of methotrexate in cases associated with a PUK episode should be closely monitored, as this severe complication may demand the use of more potent immunotherapy, such as alkylating or even anti-CD20 agents [20].

Pediatric MMP with ocular involvement is a rare condition that seems to share clinical features with the adult counterpart, albeit with a relatively more indolent evolution. Timely diagnosis and treatment are warranted but remain challenging.

## Literature search

The authors conducted an extensive literature search using the National Library of Medicine’s PubMed and Google Scholar databases for all articles published until October 2023. The following search terms were used: “mucous membrane pemphigoid”, “ocular cicatricial pemphigoid,” “pemphigoid”, “pediatric”, “childhood”, “child”, “boy”, “girl”, “infant,” “”. Case reports, case series, letters to the editor, review articles, and original articles were included. Relevant references within articles found were also included.

## Data Availability

No datasets were generated or analysed during the current study.
